# Multicenter research in dialysis centers in Brazil: recruitment and implementation of the SARC-HD study

**DOI:** 10.1590/2175-8239-JBN-2024-0009en

**Published:** 2024-12-20

**Authors:** Marvery P. Duarte, Otávio T. Nóbrega, Barbara P. Vogt, Fábio A. Vieira, Dário R. Mondini, Maryanne Z.C. Silva, Henrique S. Disessa, Rodrigo R. Krug, Bruna R.M. Sant’Helena, Daiana C. Bundchen, Maristela Bohlke, Angélica N. Adamoli, Marco C. Uchida, Carla M. Avesani, Maycon M. Reboredo, Heitor S. Ribeiro

**Affiliations:** 1 Universidade de Brasília, Faculdade de Ciências da Saúde, Brasília, DF, Brazil.; 2 Universidade Federal de Uberlândia, Faculdade de Medicina, Programa de Pós-Graduação em Ciências da Saúde, Uberlândia, MG, Brazil.; 3 Universidade Estadual de Campinas, Faculdade de Educação Física, Laboratório de Cinesiologia Aplicada, Campinas, SP, Brazil.; 4 Universidade Estadual Paulista, Faculdade de Medicina de Botucatu, Departamento de Medicina Interna, Botucatu, SP, Brazil.; 5 Universidade Estadual Paulista, Departamento de Educação Física, Bauru, SP, Brazil.; 6 Universidade de Cruz Alta, Programa de Pós-Graduação em Atenção Integral à Saúde, Cruz Alta, RS, Brazil.; 7 Faculdade IELUSC, Joinville, SC, Brasil.; 8 Universidade Federal de Santa Catarina, Programa de Pós-Graduação em Ciências da Saúde, Araranguá, SC, Brazil.; 9 Universidade Católica de Pelotas, Programa de Pós-Graduação em Saúde e Comportamento, Pelotas, RS, Brazil.; 10 Hospital de Clínicas de Porto Alegre, Serviço de Educação Física e Terapia Ocupacional, Porto Alegre, RS, Brazil.; 11 Karolinska Institutet, Department of Clinical Science, Technology and Intervention, Division of Renal Medicine and Baxter Novum, Stockholm, Suécia.; 12 Universidade Federal de Juiz de Fora, Faculdade de Medicina, Juiz de Fora, MG, Brazil.

**Keywords:** Chronic Kidney Disease, Hemodialysis, Sarcopenia, Health Information Management, Data Collection

## Abstract

**Introduction:**

Multicenter research initiatives in Brazilian dialysis centers are scarce. We described the recruitment and implementation phases of the SARC-HD study, aimed at investigating sarcopenia and its impact on adverse clinical outcomes.

**Methods:**

The SARC-HD is a cohort study being conducted with patients on hemodialysis in Brazil. The recruitment phase was defined as the period from the invitation to the center until the start of patient enrollment, whereas the implementation phase lasted from then until the completion of enrollment and baseline data collection. Upon implementation, a structured questionnaire was distributed to collect feedback from principal investigators.

**Results:**

21 centers from three Brazilian regions consented to participate, with two dropping out. Ten principal investigators oversaw the 19 sites. Nine centers (47%) were funded entirely by health insurance companies. A total of 1525 patients were screened for eligibility and 1008 were enrolled, with a 66.1% recruitment rate. Recruitment and baseline data collection took 12 [interquartile range: 5–15] weeks. Qualitative content analysis identified barriers such as a lack of infrastructure and logistics for research. Facilitators included the management and organization of the steering committee. Data collection challenges were mainly reported with the subjective 7-point global assessment and the international physical activity questionnaire. The main challenge for the ongoing maintenance phase will be the lack of standardized information in electronic health records.

**Conclusions:**

The recruitment and implementation phases of the multicenter SARC-HD study were feasible. Barriers and facilitators identified by principal investigators may help future multicenter initiatives to integrate research-related tasks into clinical routine, facilitating successful experiences.

## Introduction

Sarcopenia – an age-related condition characterized by low levels of skeletal muscle mass and physical function^1,2^ – has been recently recognized as a disease^3^. In patients with end-stage kidney disease on hemodialysis, sarcopenia is widely prevalent^4^ and has been associated with a higher mortality risk^5,6^. Even though Brazil has an outstanding number of studies that investigate sarcopenia prevalence and outcomes in this population^4,5^, a survey in Brazilian dialysis centers showed that only 37% routinely included sarcopenia-related assessments^7^. Moreover, most of the previous Brazilian studies were single-center^4,5^, and nationally representative data about the prevalence of sarcopenia in patients on hemodialysis are not yet available.

Data from the Brazilian dialysis survey in 2022 showed different estimated prevalence rates of patients on dialysis across regions^8^, in line with other major nationwide disparities in social, cultural, ethnic, and economic aspects^9^. Such diversity may limit multicenter studies to be conducted in dialysis centers, preventing public policies from benefiting from robust and representative evidence^10^. Limited government research funding in recent years in Brazil^11,12^ and the crisis in the public and private health systems^13^, especially related to dialysis^14^, may have restrained national collaborative initiatives. To the best of our knowledge, only a few large multicenter studies have been conducted in nephrology centers in Brazil (*e.g.*, ADHERE^15^, BRAZPD I^16^ and II^17^, HDFIT^18^, and COVID-19 HD-Brazil^19^), but none of them investigated sarcopenia or musculoskeletal health.

Given the need to conduct large representative multicenter research in Brazilian dialysis centers and the lack of studies exploring sarcopenia nationally, we designed the **SARC-HD** (***SARC*** *openia trajectories and their association with adverse clinical outcomes in patients on* ***H*** *emo* ***D*** *ialysis)* study, which aims to investigate the trajectories of sarcopenia and their impact on adverse clinical outcomes. Therefore, the objective of this report was to describe the recruitment and implementation phases of the study.

## Methods

### Study Design, Setting, and Procedures

The SARC-HD study protocol has been previously published, and detailed information can be found elsewhere^20^. Briefly, the SARC-HD is a multicenter, observational cohort study with a prospective design that enrolled patients undergoing hemodialysis treatment across dialysis centers from different regions in Brazil from October 2022 to April 2023. The main objectives of the study are to investigate the trajectories of sarcopenia and their impact on adverse clinical outcomes. The study design started in January 2021 from regular meetings among the leading researchers (HSR, MPD, and MMR). The study protocol followed the ethical principles of the Declaration of Helsinki and was approved by the Ethics Committees of the ICESP University Center (approval protocol on May 19, 2022, no. 5.418.365). All other institutional review boards reviewed and agreed with the approval letter. The SARC-HD study is also registered at the *Registro Brasileiro de Ensaios Clínicos* (ReBEC) platform (RBR-82p87rq).

### Data Collection

The SARC-HD study has three time points (see **Supplementary Figure 1**), as follows: recruitment and baseline assessment, 12-month follow-up and reassessment, and 24-month follow-up. At baseline, sarcopenia-related variables (*i.e.* handgrip strength, five sit-to-stand time, calf circumference, and gait speed) were objectively measured. Questionnaires were also applied to assess the risk of sarcopenia (SARC-F), levels of physical activity (international physical activity questionnaire [IPAQ] short-form), malnutrition (7-point subjective global assessment [7p-SGA]), and cognitive function (mini-mental state exam [MMSE]). Clinical (comorbidities, CKD etiology, etc.) and demographic data were collected from electronic health records. In this report, we describe the first time point. For data collection, no financial support or payment for procedures was provided, except in cases where graduate and postgraduate students received scholarships.

### SARC-HD Study Committees

**Figure 1.**
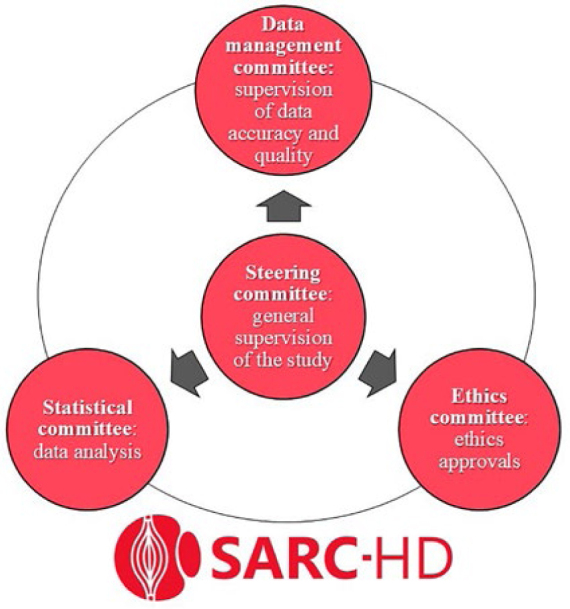
SARC-HD study committees.

Committees were established to support principal investigators and lead coordinators with the management of the study. Each committee had specific tasks and responsibilities, which are shown in Figure 1. The steering committee is responsible for the general supervision of the study, consensus meetings, staff training and standardization of methods, recruitment of additional centers, development of manual operating procedures, fund- and financial-raising, and analysis of scientific productions of the study. The data management committee is responsible for the periodic supervision of data accuracy and quality. The statistical committee is responsible for data cleaning and analysis, while the ethics committee is responsible for providing and validating all necessary documentation for each center to obtain ethical approval.

Before the beginning of the study, one principal investigator (the person with the highest academic degree and experience) and one lead coordinator (the person with the most availability for data collection supervision) were appointed to each site (full list is shown in **Supplemental Material 1**).

### Recruitment Phase

#### Dialysis centers

Recruitment of dialysis centers took place from January to August 2022 through online meetings. The steering committee (HSR, MPD, and MMR) invited members associated with the *Grupo Brasileiro de Reabilitação em Nefrologia* (Brazilian Nephrology Rehabilitation Group)^21^, but also through expanded network. If the dialysis center was associated with an academic hospital, the ethics committee of the SARC-HD study provided all necessary documentation to obtain local ethical approval through Plataforma Brazil, the Brazilian national ethics platform, linked to the originally approved submission. Centers not associated with universities were included in the originally approved submission as participating centers.

#### Patients on hemodialysis

Recruitment and baseline evaluation of the patients took place from October 2022 to May 2023. Patients aged ≥ 18 years and undergoing maintenance hemodialysis for at least three months were eligible for participation. Exclusion criteria included physical limitations, limb amputations, or other abnormalities that would interfere with physical function assessments, and medical contraindications. Each dialysis center adopted the best possible recruitment strategy, as clinical and research routines were distinct. Invitations were carried out individually by the lead coordinator or another member of the group at each center.

#### Implementation phase

The implementation phase was defined as the period between recruitment (from the first enrollment and beginning of baseline data collection) and maintenance (last assessment of included patients and beginning of the follow-up period) phases. Monthly online meetings via Google^®^ Meet with all principal investigators and lead coordinators of each site were conducted to assist in this phase by discussing previous, ongoing, and subsequent steps (see **Supplementary Table 1** for all procedures). The meetings were scheduled on the day and time most voted in an online poll (doodle.com). The steering committee (HSR, MPD, and MMR) created standardized materials with the assistance of tutorials and videos. These materials had step-by-step information and descriptions about each variable of interest. Full content is available in Brazilian Portuguese in **Supplementary Link 1** (drive.google.com/drive/folders/1mE_lx8dbG1Jw21Qtr1KyAWQLYn-_NKGx?usp=sharing). The lead coordinator at each dialysis center was responsible for training the local research team.

#### Feedback questionnaire

After the implementation phase, an online question naire was designed and shared with principal investigators to assess their perceptions about participating in the SARC-HD study. The questionnaire included information on sociodemographic, professional experience, dialysis center, barriers and facilitators, and desired output (see **Supplementary Material 2**).

#### Maintenance phase

The maintenance phase consists of the continuous follow-up for adverse clinical outcomes over 24 months and the reassessment of sarcopenia after 12 months. After the implementation phase, the data management committee independently verified and audited all baseline data to ensure the quality and accuracy of the collected data. Additionally, online meetings with all principal investigators and lead coordinators of each site are held every three months to assist with follow-up of clinical outcomes. In the maintenance phase, auditing for follow-up data will also be conducted every six months.

#### Data analysis

The quantitative data was synthesized, described using frequency and proportion, and presented in tables along with a narrative summary. Free text responses from the feedback questionnaire were qualitatively interpreted using the content analysis technique proposed by Mynaio^22^. To preserve the anonymity of the principal investigators (study participants), they were identified by codes (PI1, PI2, PI3, etc.). Moreover, responses were quoted when appropriate to assist the qualitative interpretation. For quantitative analysis, we used the Statistical Package for the Social Sciences (version 29.0, IBM Corp., Armonk, NY, USA).

## Results

### Recruitment Phase

#### Dialysis centers

**Figure 2.**
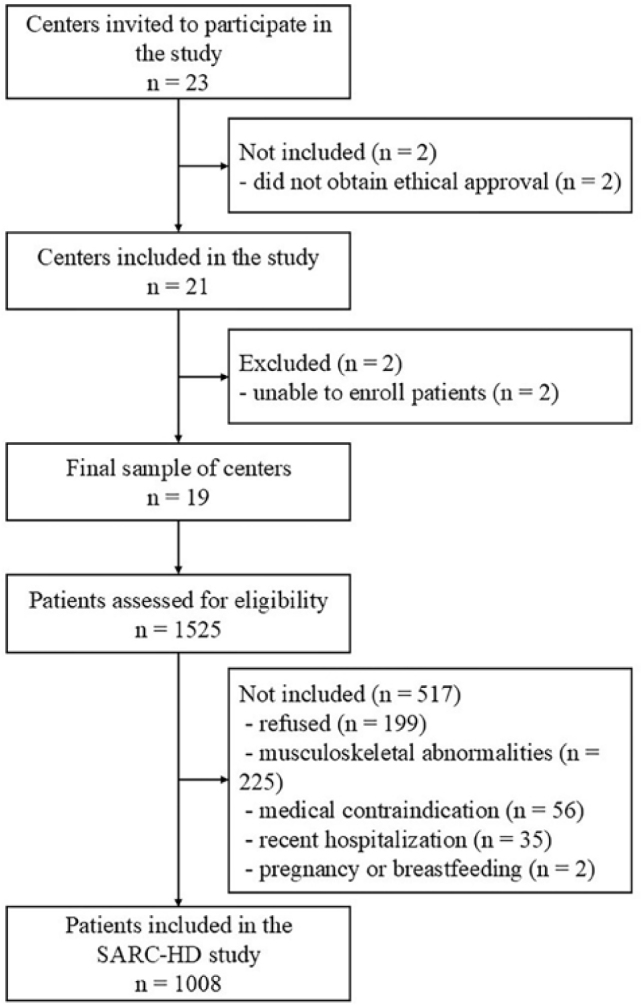
Flowchart of sample recruitment.

**Figure 3.**
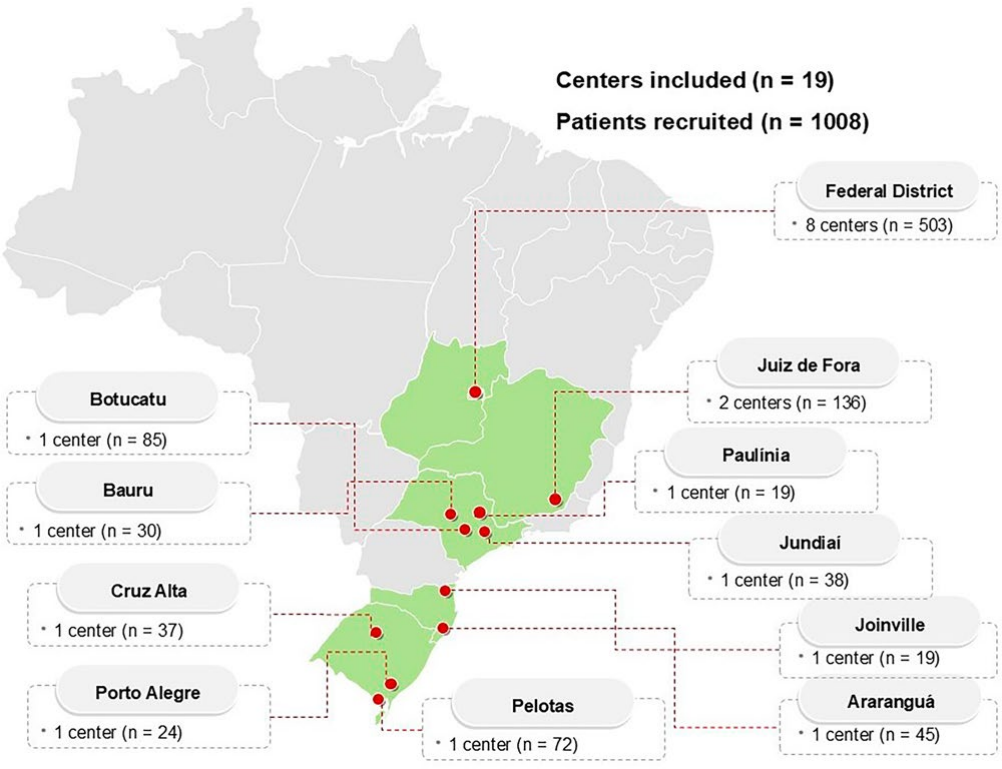
Map of Brazil with the dialysis centers included in the SARC-HD study.

Twenty-three centers were invited to participate in the SARC-HD study; two centers did not obtain ethical approval within the allotted time due to a delay in the review process, resulting in 21 centers being included in the sample. Of these, two centers were unable to enroll patients because of changes in the dialysis staff and were therefore excluded. The final sample consisted of 19 centers (Figure 2) from four states (Minas Gerais, São Paulo, Santa Catarina, and Rio Grande do Sul) and the Federal District (Figure 3), which was representative of three Brazilian regions. Of these, three centers (15.8%) associated with academic hospitals required ethical amendments to obtain local approval, while the remaining 16 centers (84.2%) were included as participating centers in the originally approved submission.

Characteristics of the dialysis centers are described in Table 1. Dialysis centers were mainly from the Mid-West region (n = 8; 42.1%), nine (47.4%) were exclusively funded by private health insurance, and 11 (57.9%) had hemodiafiltration treatment. All principal investigators had a doctorate (n = 10; 100%) and most were female (n = 6; 60%).

**Table 1.** Characteristics of the dialysis centers and principal investigators

Variables	
Patients in the center, n (median, IQR)	58 [45–97]
Patients included in the study, n (median, IQR)	45 [30–72]
Recruitment rate, % (median, IQR)	70 [56–88]
In-hospital treatment, n (%)	4 (21.1)
In-center treatment, n (%)	15 (78.9)
**Regions**, n (%)	
Mid-West	8 (42.1)
South-East	6 (31.6)
South	5 (26.3)
Is the center from a state capital? (yes)	5 (26.3)
**Dialysis modality** ^a^, n (%)	
Conventional hemodialysis	19 (100)
Hemodiafiltration	11 (57.9)
**Treatment frequency** ^a^, n (%)	
Conventional (three weekly sessions)	19 (100)
Short daily (five to six weekly sessions)	8 (42.1)
**Funding**, n (%)	
Private only (health insurances)	9 (47.4)
Public only (SUS)	3 (15.8)
Private and public	7 (36.8)
Does the center have a dietitian?	19 (100)
Does the center have an exercise professional^b^?	13 (68.4)
**Principal investigators (n = 10)^c^**	
Experience in dialysis, years (median, IQR)	8 [6–18]
Woman, n (%)	6 (60.0)
Doctorate degree, n (%)	10 (100)

SUS: Sistema Único de Saúde (Unified Health System); IQR: interquartile range.

a Sum is greater than 100% because a dialysis center can have more than one option.

b Exercise physiologists or physiotherapists.

c One principal investigator could be responsible for more than one center.

#### Patients on hemodialysis

A total of 1525 patients on hemodialysis were screened for eligibility in the 19 centers. After applying eligibility criteria and excluding refusals, the final sample consisted of 1008 patients, resulting in a 66.1% recruitment rate (Figure 2). Median recruitment rate among centers was 70% [IQR: 56 – 88]. The number of patients recruited at each center is shown in Figure 3.

#### Implementation phase

During the implementation phase, most principal investigators reported an active involvement of at least one professional from the dialysis staff team in the study (n = 17; 89.5%). All dialysis centers had graduate and/or postgraduate students involved in recruitment and data collection (n = 19; 100%).

Of the 10 principal investigators (one person could be responsible for more than one center), eight (80%) responded to the feedback questionnaire and gave additional information regarding this phase. Principal investigators and lead coordinators took a median of 2 [interquartile range (IQR): 2 – 7] weeks to train the local research teams. The recruitment and data collection took a median of 12 [IQR: 5 – 15] weeks. All principal investigators (n = 8) considered the frequency of online meetings appropriate for this phase.

Table 2 shows the qualitative content analysis of the questions regarding the implementation phase. Regarding barriers, two main categories were found: i) infrastructure and logistics; and ii) lack of adequate engagement of the dialysis staff. For the facilitators, five main categories were identified: i) patient adherence; ii) experience of the principal investigator; iii) management and organization of the steering committee; iv) engagement of dialysis staff; and v) implementation of the research as part of the clinical routine. Regarding data collection instruments, difficulties were reported in the application of the 7p-SGA, IPAQ, bioimpedance, and anthropometry. About the challenges in working on a multicenter study, two categories were described: i) attending the monthly online meetings and ii) training the research team. All free text responses (in Brazilian Portuguese) and their respective categories are shown in Supplementary Material 2.

**Table 2.** Barriers, facilitators, and experiences of the dialysis centers during the implementation phase

Questions	Categories	Main responses
**What were the main barriers experienced in data collection?**	Infrastructure and logistics	PI2: “Conduct all assessments at a single time.”
		PI5: “Logistics related to patient arrival and departure because the clinic has two floors and patients did not arrive at the same time.”
		PI6: “Infrastructure for conducting the tests […] the routine clinical practice of the dialysis centers did not include any of the study’s assessments.”
		PI8: “Restricted deadline for collecting baseline data […] evaluations of patients who depend on transportation […] these have restricted time for pre and post-HD evaluations […]”
		PI9: “[…] identification of medications used by the patients […] information in medical records did not appear to be updated […]”
	Lack of adequate engagement of the dialysis staff	PI3: “Difficulties in the engagement of some people from other areas, even with prior presentation and clarification about the project.”
		PI5: “[…] some professionals were not so helpful as to obstruct/block the progress of data collection.”
**What were the main facilitators experienced in data collection?**	Adherence of the patients	PI1: “[…] patients are already used to participating in research-related activities […]”
		PI9: “Patients knew part of the team, which made it easier to adhere to data collection […]”
	Experience of the PI	PI7: “I already have experience in collecting such variables as well as working with this population.”
	Management and organization of the steering committee	PI5: “[…] documents and spreadsheets prepared by the scientific coordination.”
		PI8: “[…] the meetings and the support of the steering committee […] in the guidance and forwarding of some processes that arose during the process; […] organization of forms, assessment instruments, and materials for data collection.”
		PI9: “Tutorials and meetings with the steering committee […]”
	Engagement of the dialysis staff	PI6: “Receptivity from the coordinators of the hemodialysis center”.
		PI8: “[…] having the partnership of my colleague from the nutrition team in the research team was very helpful […]. Support from colleagues in the medical and nursing team in organizing the schedules of patient arrival and departure for assessments […]”
	Conduct the research as part of the routine clinical practice	PI8: “Conducting the research as part of the clinical routine, with professionals and interns who work in the daily care of the patient.”
**Regarding data collection instruments, was there any difficulty?**	7p-SGA	PI1: “…We did not administer the nutrition questionnaire (*7p-SGA*).”.
		PI4: “7p-SGA”.
		PI8: “The main barrier was understanding the correct application of the AGS-7, for sure! Even with the tutorial, the questionnaire is difficult and may create many biases […] we consider that these data, in general, may not be so reliable…”
		PI9: “7p-SGA – application”.
	IPAQ	PI2: “The IPAQ was the one we had the most difficulty in applying because patients were uncertain about how to measure their daily activities.”
		PI7: “Only with the IPAQ, patients couldn’t respond adequately; data appeared inconsistent regardless of how they were approached. …”
		PI8: “IPAQ - It’s always challenging to assess activities in a typical week, considering HD and non-HD days […] we had to revisit the assessment of this instrument with different patients.”
	Bioimpedance	PI6: “[…] initially, we tried to include bioimpedance analysis. However, the unavailability of space and time for patients after the dialysis session made it unfeasible.”
		PI8: “[…] bioimpedance - Time required for post-HD session evaluation. Not having a bioimpedance device and electrodes in the unit (had to be borrowed from another department […])”.
	Anthropometry	PI9: “Anthropometry - rushed execution due to some patients wanting to leave, but we managed to perform it adequately […]”
**What were the main challenges of working in a multicenter study?**	Participating in the monthly meetings	PI6: “Participation of all centers in the meetings.”
		PI7: “[…] the different schedules as well as the days and/or times of the meetings.”
		PI9: “Adjust the time of the meetings […]”
	Training the research team	PI9: “[…] properly training the data collection team to ensure the most reliable information possible.”

7p-SGA: 7-point subjective global assessment; HD: hemodialysis; IPAQ: international physical activity questionnaire short-form; PI: principal investigator.

#### Maintenance phase

The maintenance phase is currently ongoing. About the barriers of this phase (*i.e.*, follow-up for outcomes and sarcopenia reassessment at 12 months), two main categories were found: i) lack of standardized information in electronic health records; and ii) limited research team. Principal investigators responded: “P9: *Lack of standardized information in electronic health records about the outcomes, especially length of hospitalization and mortality cause*.” and “P1: *Our research team will be limited because some students will no longer have scholarships […]*”.

**Figure 4.**
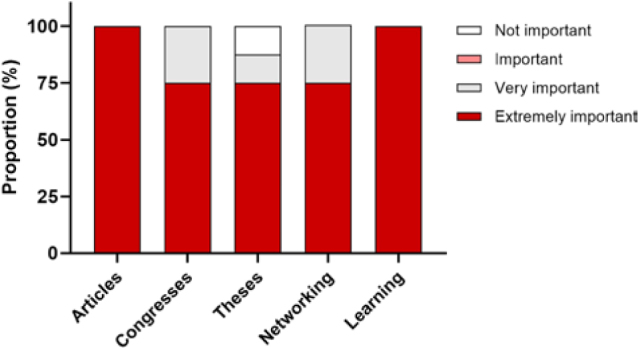
Expectations on participating in the SARC-HD study.

Figure 4 shows that when asked about their expectations of participating in the SARC-HD study, all principal investigators considered the *publication of scientific articles* and *learning/exchanging knowledge* to be extremely important.

## Discussion

In this report, we described the recruitment and implementation phases of a sarcopenia-related multicenter study in Brazilian dialysis centers. We found the SARC-HD study to be feasible, as most invited centers were able to participate and complete the implementation phase. Regarding the perceptions over this phase, the management and organization of the steering committee (*e.g.*, development of standardized tutorials and materials), and adherence of the patients were useful facilitators. On the other hand, the lack of infrastructure and logistics for research, and the lack of adequate engagement of the dialysis staff were the main categories of barriers. All principal investigators considered the publication of scientific articles and the learning experience as extremely important outputs of participating in the SARC-HD study. For the ongoing maintenance phase, the main challenge will be the lack of standardized information in electronic health records.

Multicenter collaborative networks have an important role in knowledge growth since they can recruit and enroll a larger number of participants^23^. Therefore, multicenter research enables larger studies to be conducted over a shorter period of time, improving the generalizability of the results and allowing the inclusion of geographically representative populations^24,25^. In the SARC-HD study, we were able to achieve such characteristics, engaging three Brazilian regions and more than a thousand patients. Facilitating factors were found while conducting this multicenter research, such as the development of standardized tutorials/materials and, most importantly, the commitment and continuous contact with all study sites through monthly online meetings with the principal investigators and lead coordinators.

Previous multicenter experiences in dialysis centers have been reported in Brazil (*e.g.* BRAZPD I^16^ and II^17^, HDFIT^18^, and COVID-19 HD-Brazil^19^). The HDFIT trial, for instance, invited 14 outpatient dialysis centers for a 12-month randomized clinical trial^18^. For the management of the study, a steering committee was appointed to approve the research design, protocol, addendums and changes, data analyses, etc. This has been incorporated into the SARC-HD study to allow adequate management throughout the recruitment, implementation, and maintenance phases.

Several inherent challenges are faced when conducting multicenter research, including sustained commitment, leadership, rigorous quality assurance, and careful planning, apart from regulatory and administrative aspects^24^. Marsicano-Souza et al.^26^ have previously reported the management strategies for implementing a multicenter cross-sectional study in Brazilian kidney transplantation centers (ADHERE Brazil study). They discussed that the coordination of activities was divided into areas and each area had a coordination group, which helped to collect high-quality data. Thus, we designed the study committees in the SARC-HD study to minimize such challenges and achieve high-quality data collection. Regarding other barriers, we observed difficulties in the application of certain questionnaires such as the IPAQ and the 7p-SGA. Thus, we suggest that these questionnaires should be applied by experienced professionals. Despite these challenges and barriers, the multicenter design of the SARC-HD study is in line with the goals of the National Network of Clinical Research in Brazil, which aims to support the exchange of knowledge among researchers^27^.

The experiences with the SARC-HD study may have significant clinical applicability for collaborative networks that wish to conduct multicenter research, especially in low- and middle-income countries. Despite the limited research resources and funding in countries such as Brazil, we showed that multicenter research may be feasible when properly designed. In the case of the SARC-HD study, low-cost and easy-to-apply tests were selected to have the highest possible external and ecological validity. This may have facilitated the operationalization and sustainability of the implementation phase. Therefore, we hope that these findings will support and encourage Brazilian dialysis centers to design and conduct multicenter research.

The limitations in this report are worth mentioning. Face-to-face meetings and trainings could not be conducted with all principal investigators and coordinators to ensure proper comprehension. Thus, future multicenter studies should establish local/regional committees to promote such in-person meetings if enough funding is available. The questionnaire with free text responses from the principal investigators about their experience in participating in the implementation phase of the study was not properly designed to allow a robust qualitative analysis. Future studies aimed at assessing barriers, facilitators, and perceptions should adopt validated questionnaires (if available) and/or semi-structured interviews. Despite our large sample size from three regions of Brazil, our study sample may not be sufficiently representative of the country’s dialysis center population, as differences in dialysis modality and funding source are relevant compared to recent data from the Brazilian dialysis survey in 2022^8^.

## Conclusion

We may conclude that the recruitment and implementation phases of the SARC-HD multicenter study were feasible, as most centers successfully completed both phases. The management and organization of the steering committee and the engagement of the dialysis staff were pointed as useful facilitators, while the lack of adequate infrastructure for conducting research-related tasks was the main barrier. Barriers and facilitators identified by the principal investigators may help future multicenter initiatives to integrate research-related tasks into the routine clinical practice and facilitate successful experiences.

## Data Availability

Data is available upon reasonable request to the corresponding author (HSR).

## Supplementary Material

The following online material is available for this article:

Supplementary Figure 1 - Schematic representation of the SARC-HD study.

Supplementary Link 1 - Standardized materials for data collection: https://drive.google.com/drive/folders/1mE_lx8dbG1Jw21Qtr1KyAWQLYn-_NKGx?usp=sharing.

Supplementary Table 1 - Schedule of study activities.

Supplementary Material 1 - Full list of the SARC-HD study members.

Supplementary Material 2 - Responses to the feedback questionnaire from the principal investigators.

## References

[1] Rosenberg Rosenberg (1997). Sarcopenia: origins and clinical relevance. J Nutr.

[2] Kirk B, Cawthon PM, Arai H, Ávila-Funes JA, Barazzoni R, Bhasin S (2024). The conceptual definition of sarcopenia: Delphi Consensus from
the Global Leadership Initiative in Sarcopenia (GLIS). Age Ageing.

[3] Anker SD, Morley JE, von Haehling S. (2016). Welcome to the ICD-10 code for sarcopenia. J Cachexia Sarcopenia Muscle.

[4] Duarte MP, Almeida LS, Neri SGR, Oliveira JS, Wilkinson TJ, Ribeiro HS (2024). Prevalence of sarcopenia in patients with chronic kidney disease:
a global systematic review and meta-analysis. J Cachexia Sarcopenia Muscle.

[5] Ribeiro HS, Neri SGR, Oliveira JS, Bennett PN, Viana JL, Lima RM (2022). Association between sarcopenia and clinical outcomes in chronic
kidney disease patients: a systematic review and
meta-analysis. Clin Nutr.

[6] Shu X, Lin T, Wang H, Zhao Y, Jiang T, Peng X (2022). Diagnosis, prevalence, and mortality of sarcopenia in dialysis
patients: a systematic review and meta-analysis. J Cachexia Sarcopenia Muscle.

[7] Duarte MP, Almeida LS, Böhlke M, Lima MR, Nóbrega OT, Ribeiro HS (2024). Sarcopenia in dialysis centers in Brazil: a survey study about
assessment and management. Rev Nutr.

[8] Nerbass FB, Lima HDN, Moura JA, Lugon JR, Sesso R (2024). Brazilian Dialysis Survey 2022. J Bras Nefrol.

[9] Instituto Brasileiro de Geografia e Estatística (2023). Censo demográfico 2022: população e domicílios - primeiros
resultados.

[10] Peters Peters (2020). Public policy studies: academic roots and practical
significance. AlMuntaqa.

[11] Angelo Angelo (2016). Brazil’s scientists battle to escape 20-year funding
freeze. Nature.

[12] Angelo Angelo (2019). Brazil’s government freezes nearly half of its science
spending. Nature.

[13] Costa Costa (2017). Brazilian healthcare in the context of austerity: private sector
dominant, government sector failing. Cien Saude Colet.

[14] Moura JA (2023). A crise humanitária da diálise no Brazil.

[15] Sanders-Pinheiro H, Colugnati FAB, Marsicano EO, De Geest S, Medina JOP (2018). Prevalence and correlates of non-adherence to immunosuppressants
and to health behaviours in patients after kidney transplantation in Brazil
– the ADHERE BRAZIL multicentre study: a cross-sectional study
protocol. BMC Nephrol.

[16] Fernandes N, Bastos MG, Cassi HV, Machado NL, Ribeiro JA, Martins G (2008). The Brazilian Peritoneal Dialysis Multicenter Study (BRAZPD):
characterization of the cohort. Kidney Int Suppl.

[17] Moraes TP, Figueiredo AE, Campos LG, Olandoski M, Barretti P, Pecoits R (2014). Characterization of the BRAZPD II cohort and description of
trends in peritoneal dialysis outcome across time periods. Perit Dial Int.

[18] Pecoits R, Larkin JW, Poli-de-Figueiredo CE, Cuvello No AL, Barra AB, Canhada S (2019). Design and methodology of the impact of HemoDiaFIlTration on
physical activity and self-reported outcomes: a randomized controlled trial
(HDFIT trial) in Brazil. BMC Nephrol.

[19] Lugon JR, Neves PDMM, Pio-Abreu A, do Nascimento MM, Sesso R (2022). Evaluation of central venous catheter and other risk factors for
mortality in chronic hemodialysis patients with COVID-19 in
Brazil. Int Urol Nephrol.

[20] Duarte MP, Pereira MS, Baião VM, Vieira FA, Silva MZC, Krug RR (2023). Design and methodology of the SARCopenia trajectories and
associations with adverse clinical outcomes in patients on HemoDialysis: the
SARC-HD study. BMC Nephrol.

[21] Andrade FP, Ribeiro HS, Krug RR, Reboredo MM (2022). Grupo Brasileiro de Reabilitação em Nefrologia
(GBREN). Biomotriz.

[22] Minayo Minayo (2014). O desafio do conhecimento: pesquisa qualitativa em saúde.

[23] Nelson C, Mori N, Ton T, Zunt J, Kochel T, Romero A (2018). Building a network for multicenter, prospective research of
central nervous system infections in South America: process and lessons
learned. eNeurologicalSci.

[24] Das Das (2022). Multicenter studies: relevance, design and
implementation. Indian Pediatr.

[25] Sprague S, Matta JM, Bhandari M, Dodgin D, Clark CR, Kregor P (2009). Multicenter collaboration in observational research: improving
generalizability and efficiency. J Bone Joint Surg Am.

[26] Marsicano-Souza EO, Colugnati FAB, Castro BBA, Van Keullen MDS, De Geest S, Sanders-Pinheiro H (2022). Management strategies for implementing a multicenter
cross-sectional study: lessons from the ADHERE Brazil study. Sao Paulo Med J.

[27] Brazil, Ministério da Saúde (2005). Rede Nacional de Pesquisa Clínica [Internet].

