# Supplementary material for: Multicenter research in dialysis centers in Brazil: recruitment and implementation of the SARC-HD study
Source: J Bras Nefrol. 2024 Dec 20;47(1):e20240009. doi: 10.1590/2175-8239-JBN-2024-0009en (PMC11755877; doi:10.1590/2175-8239-JBN-2024-0009en)
Supplement: Supplementary file 1 [file 2175-8239-jbn-47-1-e20240009-suppl1.pdf]

Braz. J. Nephrol. (J. Bras. Nefrol.)

https://doi.org/10.1590/2175-8239-JBN-2024-0009en

Supplementary Material to "Multicenter Research in Dialysis Centers in Brazil: Recruitment and Implementation of the SARC-HD Study"

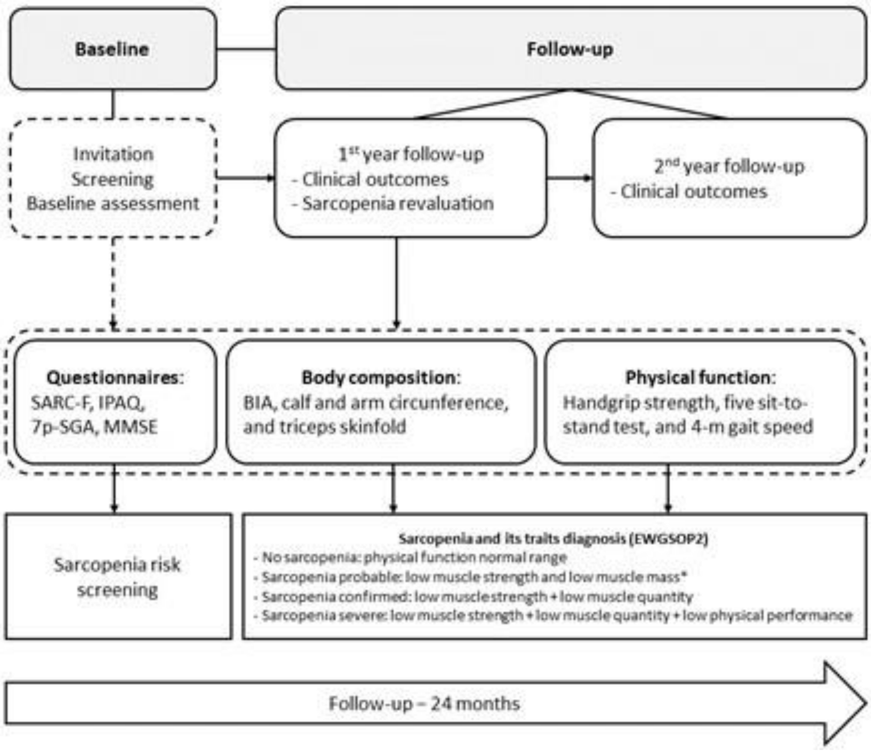

Supplementary Figure 1. Schematic representation of the SARC-HD study.

Abbreviations: 7p-SGA: 7-point subjective global assessment; BIA: bioimpedance analysis; IPA: international physical activity questionnaire; MMSE: mini-mental state exam. *Low muscle mass is also used to define probable sarcopenia.
