# Supplementary material for: Multicenter research in dialysis centers in Brazil: recruitment and implementation of the SARC-HD study
Source: J Bras Nefrol. 2024 Dec 20;47(1):e20240009. doi: 10.1590/2175-8239-JBN-2024-0009en (PMC11755877; doi:10.1590/2175-8239-JBN-2024-0009en)
Supplement: Supplementary file 2 [file 2175-8239-jbn-47-1-e20240009-suppl3.pdf]

Braz. J. Nephrol. (J. Bras. Nefrol.)

https://doi.org/10.1590/2175-8239-JBN-2024-0009en

Supplementary Material to "Multicenter Research in Dialysis Centers in Brazil: Recruitment and Implementation of the SARC-HD Study"

Supplementary Material 1. Full list of the SARC-HD Study members.

Centers (Federal District): 1) DaVita Advance, Brasília, Federal District

2) DaVita Águas Claras, Águas Claras, Federal District

3) DaVita Alvorada, Brasília, Federal District

4) DaVita Ceilândia, Ceilândia, Federal District

5) DaVita Gama, Gama, Federal District

6) DaVita JK, Taguatinga, Federal District

7) DaVita Asa Sul, Brasília, Federal District

8) DaVita Pacini, Brasília, Federal District

Principal Investigator: Heitor Ribeiro, PhD – heitorribeiro@usp.br

Coordinator: Marvery Duarte, MSc – marveryp@gmail.com

Collaborating researchers: Fábio Vieira – fabio.vieira.fisio@gmail.com; Jacqueline Monteiro, MD – Jacqueline.Monteiro-ext@davita.com; Priscila Varela – icr.priscila.fisio@gmail.com; Victor Baião, BSc – victor.baiao@aluno.unb.br; Ricardo Lima, PhD – professorricardomoreno@gmail.com; Antônio Inda-Filho, PhD – indafilho@gmail.com; Aparecido Ferreira, PhD – aparecido.ferreira@icesp.edu.br; Otávio Nóbrega, PhD – otavionobrega@unb.br

Center: NefroClass, Paulínia, São Paulo

Principal Investigator: Marco Uchida, PhD – uchida@unicamp.br

Coordinator: Dário Mondini – d233396@dac.unicamp.br

Collaborating researchers: Luiz Medina, MSc – luizmedina@hotmail.com; Luiza Sad, PT – luhsad@hotmail.com; Flávio Nishimaru, MD – flavionishi@yahoo.com.br

Center: Renal Quality, Jundiaí, São Paulo

Principal Investigator: Marco Uchida, PhD – uchida@unicamp.br

Coordinator: Dário Mondini – d233396@dac.unicamp.br

Collaborating researchers: Luiz Medina, MSc – luizmedina@hotmail.com; Luiza Sad, PT – luhsad@hotmail.com; Maria Gabriela Rosa, MD – mgabirosa@yahoo.com.br

Center: Unidade de Diálise do Hospital das Clínicas da Faculdade de Medicina de Botucatu (HCFMB), Botucatu, São Paulo

Principal Investigator: Maryanne Silva, PhD – maryanne.zilli@unesp.br

Coordinator: Maryanne Silva, PhD – maryanne.zilli@unesp.br

Collaborating researchers: Fabiana Lourenço Costa - fl.costa@unesp.br; Isabele Carolina Rodrigues - isabele.rodrigues@unesp.br; Paula Torres Presti p.presti@unesp.br; Tabata Marinda Silva tabata.silva@unesp.br

Center: DaVita Bauru, Bauru, São Paulo

Principal Investigator: Clara Rosa, PhD – clarasuemi@hotmail.com

Coordinator: Henrique Disessa, BSc – henrique.disessa@unesp.br

Collaborating researchers: None.

Centers (Juiz de Fora): 1) DaVita Juiz de Fora, Juiz de Fora, Minas Gerais

2) DaVita Rio Branco, Juiz de Fora, Minas Gerais

Principal Investigator: Maycon Reboredo, PhD – maycon.reboredo@ufjf.br

Coordinator: Marina Silveira, RD – marinasp201821@gmail.com

Collaborating researchers: Emanuele Poliana Lawall Gravina - emanueleplgravina@gmail.com, Ana Clara Cattete Bainha - anaclara_crf@hotmail.com

Center: Clínica de Nefrologia de Araranguá, Araranguá, Santa Catarina

Principal Investigator: Daiana Bundchen, PhD - daiana.bundchen@ufsc.br

Coordinator: Daiana Bundchen, PhD - daiana.bundchen@ufsc.br

Collaborating researchers: Christine Zomer Dal Molin - christine.zdm@ufsc.br, Camila Rocha Vignali - camilavignali@gmail.com, Beatriz Rocha Viana - beatrizr.viana12@gmail.com, Karine Pires Costa - karinepires01@hotmail.com, Juliana dos Santos Raimundo - juliana.sr.torres@gmail.com, Laura Polo - laurapolo2001@gmail.com, Adriane Maria Horn - adrihorn@yahoo.com.br, Lucas Alves Pizzutti - lucas.pizzutti@icloud.com, Gabrielli Vieira Carrer - gabrielli.carrer@grad.ufsc.br, Laís Corrêa de Carvalho - correalais16@gmail.com, Josué dos Santos Barbosa Júnior - j.barbosa@grad.ufsc.br, Barbara Marjorie Schwabe - babi.schwabe@gmail.com, Rafaela Aguiar Rosa - rafaelaaguiarrosa14@gmail.com,

Center: Fundação Pró-Rim, Joinville, Santa Catarina

Principal Investigator: Bruna da R. Maggi Sant'Helena, PhD – Bruna.maggi@ielusc.br

Coordinator: Bruna da R. Maggi Sant'Helena, PhD – Bruna.maggi@ielusc.br

Collaborating researchers: Rodolfo Nunes Bittencourt - rodolfo.bittencourt@ielusc.br, Maria Cecília Kohler Panno - maria.kohler@ielusc.br

Center: Unidade de Terapia Renal Jorge Bandarra Westphalen do Hospital São Vicente de Paulo, Cruz Alta, Rio Grande do Sul

Principal Investigator: Rodrigo Krug, PhD – rkrug@unicruz.edu.br

Coordinator: Rodrigo Krug, PhD – rkrug@unicruz.edu.br

Collaborating researchers: Paulo Ricardo Moreira, PhD - pmoreira@unicruz.edu.br, Thais Severo Dutra - thais.severo@hotmail.com, Jailton Possebom Marsola - jailtonpm17k@gmail.com , Anny Beatriz Somavilla- annysomavilla2002@gmail.com , Eduarda

Martins Machado - eduarda1109@hotmail.com , Taiene Rodrigues - taienerodrigues1508@gmail.com , Amiria Teixeira Santana - lordamadeu00@gmail.com .

Center: Unidade de Hemodiálise do Hospital de Clínicas de Porto Alegre (HCPA), Porto Alegre, Rio Grande do Sul

Principal Investigator: Angélica Adamoli, PhD – aadamoli@hcpa.edu.br

Coordinator: Angélica Adamoli, PhD – aadamoli@hcpa.edu.br

Collaborating researchers: Catiussa Colling - ccolling@hcpa.edu.br, Rodrigo Jacobsen, Sabrina Rodrigues da Silva - srdsilva@hcpa.edu.br, Deise dos Santos Farias - dsfarias@hcpa.edu.br , Júlia Rodrigues - jborodrigues@hcpa.edu.br , Raíssa Teixeira - araissateixeira@gmail.com .

Center: Hospital Universitário São Francisco, Pelotas, Rio Grande do Sul

Principal Investigator: Maristela Bohlke, PhD – maristela.bohlke@ucpel.edu.br

Coordinator: Maristela Bohlke, PhD – maristela.bohlke@ucpel.edu.br

Collaborating researchers:
