# Supplementary material for: Multicenter research in dialysis centers in Brazil: recruitment and implementation of the SARC-HD study
Source: J Bras Nefrol. 2024 Dec 20;47(1):e20240009. doi: 10.1590/2175-8239-JBN-2024-0009en (PMC11755877; doi:10.1590/2175-8239-JBN-2024-0009en)
Supplement: Supplementary file 3 [file 2175-8239-jbn-47-1-e20240009-suppl2.pdf]

*Braz. J. Nephrol. (J. Bras. Nefrol.)*

https://doi.org/10.1590/2175-8239-JBN-2024-0009en

# Supplementary Material to "Multicenter Research in Dialysis Centers in Brazil: Recruitment and Implementation of the SARC-HD Study"

**Supplementary Table 1.** Schedule of study activities.

| Measure | Screening | Baseline | 12 months | 24 months |
|---|---|---|---|---|
| Informed consent form | x | | | |
| Eligibility criteria | x(1) | | | |
| Demographics | x(1) | | | |
| Medical history | x | | | |
| IPAQ questionnaire | | x(2) | | |
| Mini-Mental State Exam (MMSE) | | x(2) | | |
| Nutritional assessment (7p-SGA) | | x(2) | x(2) | |
| SARC-F and SARC-CalF | | x(2) | x(2) | |
| ***Primary outcomes*** | | | | |
| Handgrip strength | | x(3) | x(3) | |
| Five-time sit-to-stand test | | x(3) | x(3) | |
| Gait speed 4-m | | x(3) | x(3) | |
| Muscle mass | | x(4) | x(4) | |
| ***Secondary outcomes*** | | | | |
| Falls | | | x | x |
| Hospitalization | | | x | x |
| Mortality | | | x | x |

[1]After signing the informed consent form; [2]The application of questionnaires was conducted DURING hemodialysis session; [3]Assessment of physical function was conducted BEFORE hemodialysis and the mid-week session; [4]Assessment of body composition was conducted AFTER hemodialysis and the mid-week session.
Abbreviation: 7p-SGA: 7-point subjective global assessment; IPAQ: international physical activity questionnaire; MMSE: mini-mental state exam.
