# Supplementary material for: Multicenter research in dialysis centers in Brazil: recruitment and implementation of the SARC-HD study
Source: J Bras Nefrol. 2024 Dec 20;47(1):e20240009. doi: 10.1590/2175-8239-JBN-2024-0009en (PMC11755877; doi:10.1590/2175-8239-JBN-2024-0009en)
Supplement: Supplementary file 4 [file 2175-8239-jbn-47-1-e20240009-suppl4.pdf]

Braz. J. Nephrol. (J. Bras. Nefrol.)

https://doi.org/10.1590/2175-8239-JBN-2024-0009en

Supplementary Material to "Multicenter Research in Dialysis Centers in Brazil: Recruitment and Implementation of the SARC-HD Study"

Supplementary Material 2. Responses to the feedback questionnaire from the principal investigators.

Barreiras

PI	Resposta	Categoria
PI1	Aplicar os testes físicos	Logística e infraestrutura
PI2	Realizar todas as avaliações em um único momento	Logística e infraestrutura
PI3	Envolvimento de algumas pessoas de outras áreas. Mesmo com apresentação e esclarecimentos prévios sobre o projeto	Engajamento da equipe de saúde com o projeto.
PI4	Liberação dos pacientes pelo nefrologista, troca de nefrologista na instituição	Engajamento da equipe de saúde com o projeto.
PI5	Logística relacionada a chegada e saída dos pacientes devido a clínica possuir dois andares e a chegada dos pacientes não ser no mesmo horário.<br><br>Também, o pouco espaço para as avaliações.<br><br>Ademais, devido ao elevado número de pacientes, o número reduzido de estudantes e profissionais envolvidos fizeram com que a coleta fosse mais demorada que o esperado.<br><br>Ademais, alguns profissionais não foram tão solícitos a ponto de atrapalhar/embargar o andamento das coletas.	Dificuldade de aplicação dos instrumentos de pesquisa<br><br>Dificuldade de aplicação dos instrumentos de pesquisa<br><br>Dificuldade de aplicação dos instrumentos de pesquisa<br><br>Engajamento da equipe de saúde com o projeto.
PI6	Espaço para a realização dos testes; disponibilidade de tempos dos pacientes para a realização dos testes antes da sessão de hemodiálise; a rotina clínica dos centros de hemodiálise não englobava nenhuma das avaliações do estudo.	Logística e infraestrutura
PI7	Escassez de equipe especializada, lacuna de equipamentos bem como as dificuldades impostas pelo centro de diálise (prazo curto para poder coletar no local).	Logística e infraestrutura
PI8	Demora na aprovação do projeto para início da coleta de dados.<br><br>Prazo restrito de coleta dos dados baseline;<br><br>Avaliações de pacientes que dependem de transporte público intermunicipal (Vans, micro-ônibus) ou que dependem de terceiros - Estes possuem horário restrito realizarem avaliações pré e pós HD. Comumente ocorrem atrasos para chegar a HD, dificultando realizar os testes físicos. Similar acontecia com a avaliação de bioimpedância, a maioria dos pacientes não conseguia ficar mais 30 minutos após a HD para avaliações da composição corporal. Para realizarem essas avaliações tinham de fazer uma reorganização da rotina no dia da coleta. Foram necessários muito estimulo motivacional, conscientização, organização prévia, ajustes e remarcações frente a imprevistos;<br><br>- Projeto de pesquisa de avaliação da sarcopenia respiratória cujas avaliações estavam acontecendo paralelamente a coleta do SARC_HD - Pacientes realizaram avaliações de forma duplicada, além de outros testes nesta pesquisa que demandavam mais tempo no hospital fora da hemodiálise, fato que causou desconfortos para pacientes e equipe de coleta. Esta pesquisa que está acontecendo paralelamente ao SARC-HD é realizada por grupo de pesquisa que não faz parte da equipe assistencial. Essa situação foi levada a chefia da unidade, com intuito de pensar em estratégias como compartilhamento de banco referente a avaliações para otimizar recursos, coleta e especialmente diminuir demandas para pacientes. A Chefia da unidade conversou com a pesquisadora responsável pelo projeto supracitado e ela justificou que o desfecho do estudo e fluxo de coleta da pesquisa é distinto o que inviabilizava o trabalho compartilhado.	Tramites éticos da pesquisa.<br><br>Dificuldade de aplicação dos instrumentos de pesquisa.<br><br>Dificuldade de aplicação dos instrumentos de pesquisa<br><br>Dificuldade de aplicação dos instrumentos de pesquisa

	- Utilização do ASG - Escala subjetiva de difícil aplicação e fechamento da avaliação;	Dificuldade de compreensão e aplicação do instrumento ASG.
PI9	Sem dúvida a principal barreira foi o entendimento para aplicação correta do AGS-7. Mesmo com o tutorial disponível, o entendimento deste questionário é difícil e pode criar muitos vieses na aplicação por diferentes pessoas. A tentativa de sanar esta dificuldade foi pedir auxílio para a nutricionista da clínica que inicialmente se mostrou pouco receptiva mas ao final, auxiliou na aplicação do questionário em alguns pacientes e treinou uma das alunas para a aplicação do mesmo. Porém, consideramos que estes dados, no geral, possam não ser tão confiáveis.<br><br>Identificação dos medicamentos utilizados pelos pacientes, as informações nos prontuários pareciam não estar atualizadas.<br>A realização da antropometria pós diálise, pois alguns pacientes estavam ansiosos para irem embora.	Dificuldade de compreensão e aplicação do instrumento ASG.<br><br>Dificuldade de aplicação dos instrumentos de pesquisa

Facilitadores

PI	Resposta	Categoria
**PI1**	O fato de os pacientes já estarem acostumados a participarem das pesquisas propostas desde o ano de 2018	A adesão dos pacientes
**PI2**	Boa adesão dos pacientes a pesquisa	A adesão dos pacientes
**PI3**	O envolvimento e interesse do pesquisador principal, Marvery	O envolvimento e experiência do PI
**PI4**	Possibilidade de melhorar a qualidade de vida dos participantes	?? Desconsiderado pois não respondeu ao objetivo da pergunta.
**PI5**	A realização de uma visita prévia à clínica antes do início da coleta de dados.<br><br>Documentos e planilhas elaboradas pela coordenação científica.	Visita prévia a clinica antes da coleta de dados.<br><br>A ótima organização dos pesquisadores principais do projeto
**PI6**	Receptividade dos pacientes e dos coordenadores do centros de hemodiálise.	Adesão dos pacientes<br><br>Engajamento da equipe de saúde.
**PI7**	Já possuo experiência na coleta de tais variáveis bem como trabalhar com esta população.	O envolvimento e experiência do PI
**PI8**	- Realizar a pesquisa como parte da rotina clínica, com profissionais e estagiários que trabalham na assistência ao paciente cotidianamente;<br><br>- Contar com a parceria da colega da nutrição na equipe de pesquisa, isso facilitou bastante a realização da ASG, avaliação e educação do paciente em relação ao seu estado nutricional; Apoio de colegas da equipe medica e enfermagem na organização de horários de entrada e saída de pacientes de HD para realizarem avaliações, facilitando processos.<br><br>- As reuniões do grupo SARC-HD, o apoio dos coordenadores Marvery e Heitor nas orientações e encaminhamentos de alguns processos de surgiram durante o processo; A organização prévia de fichas, instrumentos de avaliação, POP para coleta.	Aplicar a pesquisa na rotina clínica<br><br>Engajamento da equipe de saúde.<br><br>A ótima organização dos pesquisadores principais do projeto
**PI9**	Os tutoriais e as reuniões com a coordenação geral.<br><br>Nós tivemos uma equipe muito engajada para a coleta de dados. Os pacientes conheciam uma boa parte da equipe, o que facilitou na adesão à coleta de dados. Centro pequeno, o qual facilitou para que a coleta ocorresse efetivamente em praticamente 4 dias.	A ótima organização dos pesquisadores principais do projeto<br><br>Adesão dos pacientes

Relacionado aos instrumentos de coleta de dados, houve alguma dificuldade na aplicação, avaliação e/ou execução? Se sim, descreva.

PI	Resposta	Categoria
**PI1**	Sim, não aplicamos o questionário de nutrição.	Questionário ASG-7p

**PI2**	O questionário sobre o nível de atividade física (IPAQ) foi o que mais tivemos dificuldade na aplicação, pelo fato dos pacientes terem dificuldades para mensurar as atividades realizadas	Questionário IPAQ
**PI3**	Foi facilmente adaptado para nossa prática clínica.	--
**PI4**	Sim, asg7	Questionário ASG-7p
**PI5**	Sim. Apenas com o questionário ASG-7p	Questionário ASG-7p
**PI6**	Sim. Inicialmente tentamos incluir a bioimpedância. Entretanto, a indisponibilidade de espaço e tempo dos pacientes após a hemodiálise inviabilizou a sua realização.	Bioimpedância.
**PI7**	Apenas no IPAQ, pacientes não conseguiam responder adequadamente, dados se mostravam inconsistentes independente da maneira como eram abordados. Devido a isso, optei em não incluir os dados. As demais dificuldades se deveram aos próprios pacientes, que frequentemente se recusavam a realizar determinadas avaliações mesmo sendo previamente explicadas e agendadas.	Questionário IPAQ
**PI8**	ASG - Escala subjetiva de difícil aplicação e fechamento da avaliação;<br><br>IPAQ - Sempre desafiador avaliar as atividades em uma semana habitual, considerando dias de HD e não HD, e os participantes compreenderem que as atividades físicas em casa e deslocamento só são consideradas a partir de 10 min consecutivos. Treinar os estudantes para essa aplicação é desafiadora, tivemos que voltar a avaliação desse instrumento com diferentes pacientes.<br><br>BIA - Tempo necessário para avaliação pós sessão de HD (geralmente pós 12h ou 18hs). Não possuir um aparelho de BIA e eletrodos na unidade (necessário buscar em outro setor, material compartilhado com outras equipes, necessidade de comunicação continua com outros setores para viabilizar esses compartilhamento, mesmo com agendamento prévio).	Questionário ASG-7p<br><br>Questionário IPAQ<br><br>Bioimpedância
**PI9**	Já citados no item principais barreiras:<br>1. AGS-7 - aplicação<br>2. Antropometria - execução pela pressa de alguns paciente em irem embora, mas conseguimos realizar adequadamente.	Questionário ASG-7p<br>Antropometria

Quais foram os principais desafios enfrentados ao trabalhar com uma equipe multicêntrica?

PI	Resposta	Categoria
**PI1**	Nenhuma	Nenhuma
**PI2**	Alinhar os cronogramas e métodos de avaliações e adaptá-los as rotinas de cada unidade.	
**PI3**	O principal foi convencer as equipes fora do estudo da importância do projeto.	Não respondeu o objetivo da pergunta
**PI4**	Não descrevo como desafio, mas uma oportunidade de conhecer a dinâmica de outros centros, e outras vivências	Conhecer a rotina clínica e de pesquisa de outras Unidade renais.
**PI5**	Muitas vezes o não reconhecimento dá importância do estudo ou temática por aqueles que não estão familiarizados com a pesquisa científica pode ter implicado em uma maior brevidade e qualidade das coletas de dados.	Não respondeu o objetivo da pergunta.
**PI6**	Participação de todos os centros nas reuniões.	Participar das reuniões mensais
**PI7**	De momento apenas os cronogramas difusos bem como os dias de reuniões e/ou horários.	Participar das reuniões mensais
**PI8**	Acho que já respondi anteriormente.	Não respondeu o objetivo da pergunta
**PI9**	1. Ajustar os horários de reuniões.<br>2. Não necessariamente da equipe multicêntrica, mas que reflete no resultado do trabalho, foi uma preocupação nossa, treinar adequadamente a equipe de coleta de dados para que as informações fossem as mais confiáveis possíveis.	Participar das reuniões mensais<br><br>Treinar a equipe de pesquisa
