# Supplementary material for: Multicenter research in dialysis centers in Brazil: recruitment and implementation of the SARC-HD study
Source: J Bras Nefrol. 2024 Dec 20;47(1):e20240009. doi: 10.1590/2175-8239-JBN-2024-0009en (PMC11755877; doi:10.1590/2175-8239-JBN-2024-0009en)
Supplement: Translation

# Translation: Pesquisa multicêntrica em centros de diálise no Brasil: recrutamento e implementação do estudo SARC-HD

**DOI:** 10.1590/2175-8239-JBN-47-1-E20240009pt

**Authors:** Marvery P. Duarte, Otávio T. Nóbrega, Barbara P. Vogt, Fábio A. Vieira, Dário R. Mondini, Maryanne Z.C. Silva, Henrique S. Disessa, Rodrigo R. Krug, Bruna R.M. Sant’Helena, Daiana C. Bundchen, Maristela Bohlke, Angélica N. Adamoli, Marco C. Uchida, Carla M. Avesani, Maycon M. Reboredo, Heitor S. Ribeiro

**Affiliations:** 1 Universidade de Brasília, Faculdade de Ciências da Saúde, Brasília, DF, Brasil.; 2 Universidade Federal de Uberlândia, Faculdade de Medicina, Programa de Pós-Graduação em Ciências da Saúde, Uberlândia, MG, Brasil.; 3 Universidade Estadual de Campinas, Faculdade de Educação Física, Laboratório de Cinesiologia Aplicada, Campinas, SP, Brasil.; 4 Universidade Estadual Paulista, Faculdade de Medicina de Botucatu, Departamento de Medicina Interna, Botucatu, SP, Brasil.; 5 Universidade Estadual Paulista, Departamento de Educação Física, Bauru, SP, Brasil.; 6 Universidade de Cruz Alta, Programa de Pós-Graduação em Atenção Integral à Saúde, Cruz Alta, RS, Brasil.; 7 Faculdade IELUSC, Joinville, SC, Brasil.; 8 Universidade Federal de Santa Catarina, Programa de Pós-Graduação em Ciências da Saúde, Araranguá, SC, Brasil.; 9 Universidade Católica de Pelotas, Programa de Pós-Graduação em Saúde e Comportamento, Pelotas, RS, Brasil.; 10 Hospital de Clínicas de Porto Alegre, Serviço de Educação Física e Terapia Ocupacional, Porto Alegre, RS, Brazil.; 11 Karolinska Institutet, Department of Clinical Science, Technology and Intervention, Division of Renal Medicine and Baxter Novum, Stockholm, Suécia.; 12 Universidade Federal de Juiz de Fora, Faculdade de Medicina, Juiz de Fora, MG, Brazil.

**Keywords:** Doença Renal Crônica, Hemodiálise, Sarcopenia, Gestão de Informações de Saúde, Coleta de Dados

## Abstract

**Introdução:**

Pesquisa multicêntrica em centros de diálise brasileiros são escassas. Descrevemos as fases de recrutamento e implementação do estudo SARC-HD, visando investigar a sarcopenia e seu impacto em desfechos clínicos adversos.

**Métodos:**

O SARC-HD é um estudo de coorte realizado com pacientes em hemodiálise no Brasil. A fase de recrutamento foi considerada o período entre convite ao centro e início da inclusão de pacientes, enquanto a fase de implementação durou a partir de então até a conclusão do recrutamento e coleta de dados basais. Após a implementação, um questionário estruturado foi distribuído para coletar feedback dos principais pesquisadores.

**Resultados:**

21 centros de três regiões brasileiras consentiram em participar, com duas desistências. Dez pesquisadores principais supervisionaram 19 locais. Nove centros (47%) eram totalmente financiados por planos de saúde. 1525 pacientes foram avaliados para elegibilidade e 1008 foram incluídos, com taxa de recrutamento de 66,1%. Recrutamento e coleta de dados basais levaram 12 semanas [intervalo interquartil: 5–15]. A análise qualitativa de conteúdo identificou barreiras como falta de infraestrutura e logística para pesquisa. Os facilitadores incluíram gestão e organização do comitê gestor. Os desafios da coleta de dados foram relatados principalmente quanto à avaliação global subjetiva de 7 pontos e o questionário internacional de atividade física. O principal desafio para a fase de manutenção será a falta de informações padronizadas nos prontuários eletrônicos.

**Conclusões:**

As fases de recrutamento e implementação do estudo multicêntrico SARC-HD foram viáveis. Barreiras e facilitadores identificados pelos pesquisadores principais podem auxiliar futuras iniciativas multicêntricas a integrar tarefas relacionadas à pesquisa na rotina clínica, facilitando experiências bem-sucedidas.

## Introdução

A sarcopenia - uma condição relacionada à idade caracterizada por baixos níveis de massa muscular esquelética e função física^1,2^ – foi recentemente reconhecida como uma doença^3^. Em pacientes com doença renal em estágio terminal em hemodiálise, a sarcopenia é amplamente prevalente^4^ e tem sido associada a um maior risco de mortalidade^5,6^. Embora o Brasil possua um número excepcional de estudos que investigam a prevalência e os desfechos da sarcopenia nesta população^4,5^, uma pesquisa recente em centros brasileiros de diálise revelou que apenas 37% incluíam rotineiramente avaliações relacionadas à sarcopenia^7^. Além disso, a maioria dos estudos brasileiros prévios foi de centro único^4,5^, e dados representativos em nível nacional sobre a prevalência de sarcopenia em pacientes em hemodiálise ainda não estão disponíveis.

Dados do Censo Brasileiro de Diálise de 2022 mostraram diferentes taxas estimadas de prevalência de pacientes em diálise nas diversas regiões^8^, em consonância com outras grandes disparidades nacionais em aspectos sociais, culturais, étnicos e econômicos^9^. Essa diversidade pode limitar a realização de estudos multicêntricos em centros de diálise, impossibilitando que políticas públicas se beneficiem de evidências robustas e representativas^10^. O financiamento governamental limitado para pesquisa nos últimos anos no Brasil^11,12^ e a crise nos sistemas de saúde pública e privada^13^, especialmente em relação à diálise^14^, podem ter restringido as iniciativas nacionais de colaboração. Segundo nosso conhecimento, somente alguns grandes estudos multicêntricos foram realizados em centros de nefrologia no Brasil (por exemplo, ADHERE^15^, BRAZPD I^16^ e II^17^, HDFIT^18^ e COVID-19 HD-Brazil^19^), mas nenhum deles investigou a sarcopenia ou a saúde musculoesquelética.

Dada a necessidade de realizar pesquisas multicêntricas representativas e de grande porte nos centros brasileiros de diálise e a ausência de estudos que explorem a sarcopenia em âmbito nacional, delineamos o estudo **SARC-HD** - ***SARC*** *openia trajectories and their association with adverse clinical outcomes in patients on* ***H*** *emo* ***D*** *ialysis* (“Trajetórias da sarcopenia e sua associação com desfechos clínicos adversos em pacientes em hemodiálise”), que tem como objetivo investigar as trajetórias da sarcopenia e seus impactos em desfechos clínicos adversos. Portanto, o objetivo deste relato foi descrever as fases de recrutamento e implementação do estudo.

## Métodos

### Desenho, Local e Procedimentos do Estudo

O protocolo do estudo SARC-HD foi publicado anteriormente e informações detalhadas podem ser encontradas em outras fontes^20^. De forma resumida, o SARC-HD é um estudo de coorte observacional multicêntrico com um desenho prospectivo que incluiu pacientes submetidos a tratamento de hemodiálise em centros de diálise em diferentes regiões do Brasil, de outubro de 2022 a abril de 2023. Os principais objetivos do estudo são investigar as trajetórias da sarcopenia e seus impactos nos desfechos clínicos adversos. O desenho do estudo teve início em janeiro de 2021 a partir de reuniões regulares entre os principais pesquisadores (HSR, MPD e MMR). O protocolo do estudo seguiu os princípios éticos da Declaração de Helsinque e foi aprovado pelos Comitês de Ética do Centro Universitário ICESP (protocolo de aprovação em 19 de maio de 2022, n° 5.418.365). Todos os demais comitês de ética analisaram e concordaram com a carta de aprovação. O estudo SARC-HD também está registrado na plataforma Registro Brasileiro de Ensaios Clínicos (ReBEC) (RBR-82p87rq).

### Coleta de Dados

O estudo SARC-HD apresenta três períodos de avaliação (ver **Supplementary Figure 1**), a saber: recrutamento e avaliação basal, acompanhamento e reavaliação de 12 meses e acompanhamento de 24 meses. No período inicial, as variáveis relacionadas à sarcopenia (ou seja, força de preensão manual, tempo no teste de sentar-levantar cinco vezes, circunferência da panturrilha e velocidade da marcha) foram medidas objetivamente. Também foram aplicados questionários para avaliar o risco de sarcopenia (SARC-F), níveis de atividade física (questionário internacional de atividade física [IPAQ], versão curta), desnutrição (avaliação global subjetiva de 7 pontos [AGS-7p]) e função cognitiva (mini exame do estado mental [MEEM]). Dados clínicos (comorbidades, etiologia da DRC, etc.) e demográficos foram coletados a partir de prontuários médicos eletrônicos. Neste relato, descrevemos o primeiro período. Para a coleta de dados, não foi fornecido nenhum apoio financeiro ou pagamento pelos procedimentos, exceto nos casos em que os alunos de graduação e pós-graduação receberam bolsas de estudo.

### Comitês do Estudo Sarc-HD

Foram estabelecidos comitês para apoiar os pesquisadores e os coordenadores principais na gestão do estudo. Cada comitê recebeu tarefas e responsabilidades específicas, conforme mostrado na Figura 1. O comitê gestor é responsável pela supervisão geral do estudo, reuniões de consenso, treinamento de equipe e padronização de métodos, recrutamento de centros adicionais, desenvolvimento de procedimentos operacionais padrão, captação de recursos e financiamento e análise das produções científicas do estudo. O comitê de gestão de dados é responsável pela supervisão periódica da precisão e qualidade dos dados. O comitê estatístico é responsável pela limpeza e análise de dados, enquanto o comitê de ética é responsável por fornecer e validar toda a documentação necessária para que cada centro obtenha aprovação ética.

**Figura 1.** Comitês do estudo SARC-HD.

Antes do início do estudo, um pesquisador principal (a pessoa com o mais alto grau acadêmico e experiência) e um coordenador principal (a pessoa com maior disponibilidade para a supervisão da coleta de dados) foram designados para cada centro (a lista completa consta no **Supplementary Material 1**).

### Fase de Recrutamento

#### Centros de diálise

O recrutamento dos centros de diálise ocorreu de janeiro a agosto de 2022 por meio de reuniões online. O comitê gestor (HSR, MPD e MMR) convidou membros associados ao Grupo Brasileiro de Reabilitação em Nefrologia^21^, mas o convite também foi realizado por meio de uma rede ampliada. Caso o centro de diálise estivesse associado a um hospital acadêmico, o comitê de ética do estudo SARC-HD fornecia toda a documentação necessária para obter a aprovação ética local por meio da Plataforma Brasil, a plataforma nacional de ética do Brasil, vinculada à submissão originalmente aprovada. Centros não associados a universidades foram incluídos na submissão originalmente aprovada como centros participantes.

#### Pacientes em hemodiálise

O recrutamento e a avaliação basal dos pacientes foram realizados de outubro de 2022 a maio de 2023. Pacientes com 18 anos de idade ou mais, submetidos à hemodiálise de manutenção por pelo menos três meses, foram elegíveis para participação. Os critérios de exclusão incluíram limitações físicas, amputações de membros ou outras anormalidades que interferissem nas avaliações da função física, além de contraindicações médicas. Cada centro de diálise adotou a melhor estratégia de recrutamento possível, uma vez que as rotinas clínicas e de pesquisa eram distintas. Os convites foram realizados individualmente pelo coordenador principal ou por outro membro do grupo em cada centro.

#### Fase de implementação

A fase de implementação foi considerada como o período entre as fases de recrutamento (desde a primeira inclusão e o início da coleta de dados basais) e de manutenção (última avaliação dos pacientes incluídos e início do período de acompanhamento). Foram realizadas reuniões mensais on-line via Google^®^ Meet com todos os pesquisadores e coordenadores principais de cada local para auxiliar nessa fase, discutindo as etapas anteriores, em andamento e subsequentes (consulte a **Supplementary Table 1** para todos os procedimentos). As reuniões foram agendadas para o dia e horário mais votados em uma enquete online (doodle.com). O comitê gestor (HSR, MPD e MMR) criou materiais padronizados com o auxílio de tutoriais e vídeos. Esses materiais continham informações e descrições passo a passo sobre cada variável de interesse. O conteúdo completo está disponível em português brasileiro no **Supplementary Link 1** (drive.google.com/drive/folders/1mE_lx8dbG1Jw21Qtr1KyAWQLYn-_NKGx?usp=sharing). O coordenador principal de cada centro de diálise foi responsável pelo treinamento da equipe local de pesquisa.

#### Questionário de feedback

Após a fase de implementação, um questionário on-line foi elaborado e compartilhado com os pesquisadores principais para avaliar suas percepções sobre a participação no estudo SARC-HD. O questionário incluía informações sobre dados sociodemográficos, experiência profissional, centro de diálise, barreiras e facilitadores e os produtos desejados (consulte o **Supplementary Material 2**).

#### Fase de manutenção

A fase de manutenção consiste no acompanhamento contínuo de desfechos clínicos adversos ao longo de 24 meses e na reavaliação da sarcopenia após 12 meses. Após a fase de implementação, o comitê de gestão de dados verificou e auditou de forma independente todos os dados basais para garantir a qualidade e a precisão das informações coletadas. Além disso, reuniões on-line com todos os pesquisadores e coordenadores principais de cada local são realizadas a cada três meses para auxiliar no acompanhamento dos desfechos clínicos. Na fase de manutenção, a auditoria para dados de acompanhamento também será realizada a cada seis meses.

#### Análise de dados

Os dados quantitativos foram sintetizados, descritos por meio de frequência e proporção e apresentados em tabelas, acompanhados de um resumo narrativo. As respostas abertas do questionário de feedback foram interpretadas qualitativamente utilizando a técnica de análise de conteúdo proposta por Mynaio^22^. Para preservar o anonimato dos pesquisadores principais (participantes do estudo), eles foram identificados por códigos (PP1, PP2, PP3, etc.). Além disso, as respostas foram citadas quando apropriado para auxiliar na interpretação qualitativa. Para a análise quantitativa, utilizamos o *Statistical Package for the Social Sciences* (versão 29.0, IBM Corp., Armonk, NY, EUA).

## Resultados

### Fase de Recrutamento

#### Centros de diálise

Vinte e três centros foram convidados a participar do estudo SARC-HD; dois centros não obtiveram aprovação ética dentro do prazo estipulado devido a um atraso no processo de revisão, o que resultou na inclusão de 21 centros na amostra. Destes, dois centros não puderam inscrever pacientes devido a alterações na equipe de diálise e foram, portanto, excluídos. A amostra final foi composta por 19 centros (Figura 2) de quatro estados (Minas Gerais, São Paulo, Santa Catarina e Rio Grande do Sul) e do Distrito Federal (Figura 3), representativos de três regiões brasileiras. Destes, três centros (15,8%) associados a hospitais acadêmicos precisaram de emendas éticas para obter aprovação local, enquanto os demais 16 centros (84,2%) foram incluídos como centros participantes na submissão originalmente aprovada.

**Figura 2.** Fluxograma do recrutamento de amostras.

**Figura 3.** Mapa do Brasil com os centros de diálise incluídos no estudo SARC-HD.

As características dos centros de diálise estão descritas na **Tabela 1**. Os centros de diálise eram principalmente da região Centro-Oeste (n = 8; 42,1%), nove (47,4%) atendiam somente pacientes com plano de saúde privado e 11 (57,9%) dispunham de tratamento de hemodiafiltração. Todos os pesquisadores principais possuíam doutorado (n = 10; 100%) e a maioria era do sexo feminino (n = 6; 60%).

**Tabela 1:** Características dos centros de diálise e dos pesquisadores principais

Variáveis		
Pacientes no centro, n (mediana, IIQ)	58 [45–97]
Pacientes incluídos no estudo, n (mediana, IIQ)	45 [30–72]
Taxa de recrutamento, % (mediana, IIQ)	70 [56–88]
Tratamento hospitalar, n (%)	4 (21,1)
Tratamento no centro, n (%)	15 (78,9)
**Regiões**, n (%)	
Centro-Oeste	8 (42,1)
Sudeste	6 (31,6)
Sul	5 (26,3)
O centro está localizado em uma capital de estado? (sim)	5 (26,3)
**Modalidade de diálise** ^a^, n (%)	
Hemodiálise convencional	19 (100)
Hemodiafiltração	11 (57,9)
**Frequência do tratamento** ^a^, n (%)	
Convencional (três sessões semanais)	19 (100)
Curta duração diária (cinco a seis sessões semanais)	8 (42,1)
**Financiamento**, n (%)	
Somente privado (planos de saúde)	9 (47,4)
Somente público (SUS)	3 (15,8)
Privado e público	7 (36,8)
O centro possui nutricionista?	19 (100)
O centro possui profissional do exercício?^b^?	13 (68,4)
**Pesquisadores principais (n = 10)** ^c^	
Experiência em diálise, anos (mediana, IIQ)	8 [6–18]
Mulher, n (%)	6 (60,0)
Doutorado, n (%)	10 (100)

SUS: Sistema Único de Saúde; IIQ: intervalo interquartil.

a A soma é superior a 100% pois um centro de diálise pode ter mais de uma opção.

b Profissional de educação física ou fisioterapeuta.

c Um pesquisador principal poderia ser responsável por mais de um centro.

#### Pacientes em hemodiálise

Foram selecionados 1525 pacientes em hemodiálise para elegibilidade nos 19 centros. Após a aplicação dos critérios de elegibilidade e a exclusão de recusas, a amostra final foi composta por 1008 pacientes, resultando em uma taxa de recrutamento de 66,1% (Figura 2). A taxa mediana de recrutamento entre os centros foi de 70% [IIQ: 56–88]. O número de pacientes recrutados em cada centro é mostrado na Figura 3.

#### Fase de implementação

Durante a fase de implementação, a maioria dos pesquisadores principais relatou o envolvimento ativo de pelo menos um profissional da equipe de diálise no estudo (n = 17; 89,5%). Todos os centros de diálise possuíam estudantes de graduação e/ou pós-graduação envolvidos no recrutamento e na coleta de dados (n = 19; 100%).

Dos 10 pesquisadores principais (uma pessoa poderia ser responsável por mais de um centro), oito (80%) responderam ao questionário de feedback e forneceram informações adicionais sobre essa fase. Os pesquisadores e coordenadores principais dedicaram uma mediana de 2 [intervalo interquartil (IIQ): 2–7] semanas para treinar as equipes locais de pesquisa. O recrutamento e a coleta de dados levaram uma mediana de 12 [IIQ: 5–15] semanas. Todos os pesquisadores principais (n = 8) consideraram a frequência das reuniões on-line adequada para essa fase.

A **Tabela 2** apresenta a análise qualitativa do conteúdo das perguntas referentes à fase de implementação. Quanto às barreiras, foram identificadas duas categorias principais: i) infraestrutura e logística; e ii) falta de engajamento adequado da equipe de diálise. Em relação aos facilitadores, foram identificadas cinco categorias principais: i) adesão do paciente; ii) experiência do pesquisador principal; iii) supervisão e organização do comitê gestor; iv) engajamento da equipe de diálise; e v) implementação da pesquisa como parte da rotina clínica. No que diz respeito aos instrumentos de coleta de dados, foram relatadas dificuldades na aplicação do AGS-7p, IPAQ, bioimpedância e antropometria. Sobre os desafios de trabalhar em um estudo multicêntrico, foram descritas duas categorias: i) participação nas reuniões mensais on-line e ii) treinamento da equipe de pesquisa. Todas as respostas abertas (em português brasileiro) e suas respectivas categorias são apresentadas no **Supplementary Material 2**.

**Tabela 2:** Barreiras, facilitadores e experiências dos centros de diálise durante a fase de implementação

Questões	Categorias	Principais respostas
**Quais foram as principais barreiras enfrentadas na coleta de dados?**	Infraestrutura e logística	PP2: “Realizar todas as avaliações em um único momento.”
		PP5: “Logística relacionada à chegada e saída de pacientes, pois a clínica possui dois andares e os pacientes não chegavam ao mesmo tempo.”
		PP6: “Infraestrutura para a realização dos testes [...] a prática clínica de rotina dos centros de diálise não incluiu nenhuma das avaliações do estudo.”
		PP8: “Prazo restrito para a coleta de dados basais [...] avaliações de pacientes que dependem de transporte [...] esses têm tempo restrito para avaliações pré e pós-HD […]”
		PP9: “[…] identificação dos medicamentos utilizados pelos pacientes [...] as informações nos prontuários médicos não pareciam estar atualizadas […]”
	Falta de engajamento adequado da equipe de diálise	PP3: “Dificuldades no engajamento de algumas pessoas de outras áreas, mesmo com apresentação e esclarecimento prévios sobre o projeto.”
		PP5: “[…] alguns profissionais não foram tão prestativos, a ponto de obstruir/bloquear o progresso da coleta de dados.”
**Quais foram os principais facilitadores experimentados na coleta de dados?**	Adesão dos pacientes	PP1: “[…] os pacientes já estão acostumados a participar de atividades relacionadas à pesquisa […]”
		PP9: “Os pacientes conheciam parte da equipe, o que facilitou a adesão à coleta de dados […]”
	Experiência do PP	PP7: “Já tenho experiência na coleta dessas variáveis, bem como no trabalho com essa população.”
	Gestão e organização do comitê gestor	PP5: “[…] documentos e planilhas preparados pela coordenação científica.”
		PP8: “[…] as reuniões e o suporte do comitê gestor [...] na orientação e encaminhamento de alguns pontos que surgiram durante o processo; [...] organização de formulários, instrumentos de avaliação e materiais para coleta de dados.”
		PP9: “Tutoriais e reuniões com o comitê gestor […]”
	Engajamento da equipe de diálise	PP6: “Receptividade dos coordenadores do centro de hemodiálise”.
		PP8: “[…] ter a parceria do meu colega da equipe de nutrição na equipe de pesquisa foi muito útil [...]. Apoio dos colegas das equipes médica e de enfermagem na organização dos horários de chegada e saída dos pacientes para as avaliações […]”
	Realizar a pesquisa como parte da prática clínica de rotina	PP8: “Realização da pesquisa como parte da rotina clínica, com profissionais e estagiários que trabalham no atendimento diário aos pacientes.”
**Em relação aos instrumentos de coleta de dados, houve alguma dificuldade?**	AGS-7p	PP1: “Não administramos o questionário nutricional (*AGS*-*7p*).”.
		PP4: “AGS-7p”.
		PP8: “A principal barreira foi entender a aplicação correta do AGS-7, com certeza! Mesmo com o tutorial, o questionário é difícil e pode gerar muitos vieses [...] consideramos que esses dados, em geral, podem não ser tão confiáveis...”
		PP9: “AGS-7p – aplicação”.
	IPAQ	PP2: “O IPAQ foi o que tivemos mais dificuldade em aplicar, pois os pacientes estavam em dúvida sobre como mensurar suas atividades diárias.”
	IPAQ	PP7: “Somente com o IPAQ, os pacientes não conseguiram responder adequadamente; os dados pareciam inconsistentes, independentemente de como eram abordados. …”
		PP8: “IPAQ - É sempre um desafio avaliar as atividades em uma semana típica, considerando os dias de HD e os dias sem HD [...] tivemos que revisitar a avaliação desse instrumento com diferentes pacientes.”
	Bioimpedância	PP6: “[…] inicialmente, tentamos incluir a análise de bioimpedância. No entanto, a indisponibilidade de espaço e tempo para os pacientes após a sessão de diálise tornou isso inviável.”
		PP8: “[…] bioimpedância - Tempo necessário para avaliação pós-sessão de HD. Não ter um dispositivo de bioimpedância e eletrodos na unidade (foi necessário emprestá-los de outro departamento) […])”.
	Antropometria	PP9: “Antropometria - execução apressada devido ao desejo de alguns pacientes de ir embora, mas conseguimos realizá-la de forma adequada […]”
**Quais foram os principais desafios de trabalhar em um estudo multicêntrico?**	Participar das reuniões mensais	PP6: “Participação de todos os centros nas reuniões.”
		PP7: “[…] as diferentes agendas, bem como os dias e/ou horários das reuniões.”
		PP9: “Ajustar o horário das reuniões […]”
	Treinar a equipe de pesquisa	PP9: “[…] treinar adequadamente a equipe de coleta de dados para garantir as informações mais confiáveis possíveis.”

AGS-7p: Avaliação global subjetiva de 7 pontos; HD: hemodiálise; IPAQ: questionário internacional de atividade física versão curta; PP: pesquisador principal.

#### Fase de manutenção

A fase de manutenção está atualmente em andamento. Quanto às barreiras desta fase (ou seja, acompanhamento dos desfechos e reavaliação aos 12 meses), foram encontradas duas categorias principais: i) falta de informações padronizadas nos prontuários eletrônicos; e ii) equipe de pesquisa limitada. Os pesquisadores principais responderam: “P9: *Falta de informações padronizadas nos prontuários eletrônicos sobre os desfechos, especialmente tempo de permanência hospitalar e causa da mortalidade*” e “P1: *Nossa equipe de pesquisa estará limitada porque alguns alunos não receberão mais bolsas de estudo […]*”.

A Figura 4 mostra que, ao serem questionados sobre suas expectativas em relação à participação no estudo SARC-HD, todos os pesquisadores principais consideraram a *publicação de artigos científicos* e o *aprendizado/troca de conhecimentos* extremamente importantes.

**Figura 4.** Expectativas sobre a participação no estudo SARC-HD.

## Discussão

Neste relato, descrevemos as fases de recrutamento e implementação de um estudo multicêntrico relacionado à sarcopenia em centros brasileiros de diálise. Consideramos o estudo SARC-HD viável, já que a maioria dos centros convidados conseguiu participar e concluir a fase de implementação. Em relação às percepções sobre esta fase, a gestão e a organização do comitê gestor (por exemplo, o desenvolvimento de tutoriais e materiais padronizados) e a adesão dos pacientes foram facilitadores úteis. Por outro lado, a falta de infraestrutura e logística para pesquisa e a falta de engajamento adequado da equipe de diálise foram as principais categorias de barreiras. Todos os pesquisadores principais consideraram a publicação de artigos científicos e a troca de conhecimentos como resultados extremamente importantes decorrentes da participação no estudo SARC-HD. Para a fase de manutenção contínua, o principal desafio será a falta de informações padronizadas nos prontuários eletrônicos.

Redes colaborativas multicêntricas desempenham um papel importante no crescimento do conhecimento, pois podem recrutar e inscrever um maior número de participantes^23^. Portanto, a pesquisa multicêntrica permite a realização de estudos mais amplos em um período de tempo mais curto, melhorando a generalização dos resultados e possibilitando a inclusão de populações geograficamente representativas^24,25^. No estudo SARC-HD, conseguimos atingir tais características, envolvendo três regiões brasileiras e mais de mil pacientes. Foram encontrados fatores facilitadores durante a realização desta pesquisa multicêntrica, como o desenvolvimento de tutoriais/materiais padronizados e, sobretudo, o compromisso e o contato contínuo com todos os locais de estudo por meio de reuniões mensais online com os pesquisadores e coordenadores principais.

Experiências multicêntricas anteriores em centros de diálise foram relatadas no Brasil (por exemplo, BRAZPD I^16^ e II^17^, HDFIT^18^, e COVID-19 HD-Brazil^19^). O estudo HDFIT, por exemplo, convidou 14 centros de diálise ambulatorial para um ensaio clínico randomizado de 12 meses^18^. Para a organização do estudo, foi designado um comitê gestor com o objetivo de aprovar o desenho da pesquisa, o protocolo, adendos e alterações, análises de dados, etc. Isso foi incorporado ao estudo SARC-HD para permitir uma gestão adequada ao longo das fases de recrutamento, implementação e manutenção.

Diversos desafios inerentes são enfrentados durante a realização de pesquisas multicêntricas, incluindo comprometimento sustentado, liderança, garantia de qualidade rigorosa e planejamento cuidadoso, além dos aspectos regulatórios e administrativos^24^. Marsicano-Souza et al.^26^ relataram anteriormente as estratégias de gestão para a implementação de um estudo transversal multicêntrico em centros brasileiros de transplante renal (estudo ADHERE Brasil). Eles abordaram o fato de que a coordenação das atividades foi dividida em áreas, cada uma com um grupo de coordenação, o que contribuiu para a coleta de dados de alta qualidade. Assim, desenvolvemos os comitês no estudo SARC-HD para minimizar tais desafios e obter uma coleta de dados de alta qualidade. Com relação a outras barreiras, observamos dificuldades na aplicação de determinados questionários, como o IPAQ e o AGS-7p. Dessa forma, sugerimos que esses questionários sejam aplicados por profissionais experientes. Apesar desses desafios e barreiras, o desenho multicêntrico do estudo SARC-HD está em consonância com os objetivos da Rede Nacional de Pesquisa Clínica do Brasil, que visa apoiar a troca de conhecimento entre pesquisadores^27^.

As experiências com o estudo SARC-HD podem ter aplicabilidade clínica significativa para redes colaborativas que desejam realizar pesquisas multicêntricas, especialmente em países de baixa e média renda. Apesar dos recursos e financiamento limitados para pesquisa em países como o Brasil, mostramos que uma pesquisa multicêntrica pode ser viável quando planejada adequadamente. No caso do estudo SARC-HD, foram selecionados testes de baixo custo e de fácil aplicação para obter a maior validade externa e ecológica possível. Isso pode ter facilitado a operacionalização e a sustentabilidade da fase de implementação. Portanto, esperamos que esses achados apoiem e incentivem os centros de diálise brasileiros a desenvolver e realizar pesquisas multicêntricas.

As limitações do relato deste estudo merecem ser mencionadas. Não foi possível realizar reuniões e treinamentos presenciais com todos os pesquisadores e coordenadores principais para garantir a compreensão adequada. Dessa forma, os futuros estudos multicêntricos devem estabelecer comitês locais/regionais para promover essas reuniões presenciais, caso haja financiamento suficiente disponível. O questionário com respostas abertas, por parte dos pesquisadores principais, a respeito de sua experiência em participar da fase de implementação do estudo não foi adequadamente elaborado para permitir uma análise qualitativa robusta. Estudos futuros com o objetivo de avaliar barreiras, facilitadores e percepções devem adotar questionários validados (se disponíveis) e/ou entrevistas semiestruturadas. Apesar de nossa ampla amostra proveniente de três regiões do Brasil, é possível que a amostra do nosso estudo não seja suficientemente representativa da população dos centros de diálise do país, uma vez que as diferenças na modalidade de diálise e na fonte de financiamento são relevantes quando comparadas aos dados recentes do censo brasileiro de diálise de 2022^8^.

## Conclusão

Podemos concluir que as fases de recrutamento e implementação do estudo multicêntrico SARC-HD foram viáveis, uma vez que a maioria dos centros concluiu com êxito ambas as fases. A supervisão e a organização do comitê gestor e o envolvimento da equipe de diálise foram apontados como facilitadores úteis, enquanto a falta de infraestrutura adequada para a realização de tarefas relacionadas à pesquisa foi a principal barreira. As barreiras e facilitadores identificados pelos pesquisadores principais podem auxiliar futuras iniciativas multicêntricas a integrar tarefas relacionadas à pesquisa na prática clínica de rotina e facilitar experiências bem-sucedidas.

## Data Availability

Os dados estão disponíveis mediante solicitação razoável ao autor correspondente (HSR).

## Material Suplementar

O seguinte material online está disponível para o presente artigo:

Supplementary Figure 1 - Schematic representation of the SARC-HD study.

Supplementary Link 1 - Standardized materials for data collection: https://drive.google.com/drive/folders/1mE_lx8dbG1Jw21Qtr1KyAWQLYn-_NKGx?usp=sharing.

Supplementary Table 1 - Schedule of study activities.

Supplementary Material 1 - Full list of the SARC-HD study members.

Supplementary Material 2 - Responses to the feedback questionnaire from the principal investigators.
